# Association between vitamin D status and insulin resistance in Korean adolescents: differential effects of obesity using non-insulin-based indices

**DOI:** 10.1186/s12887-026-06510-5

**Published:** 2026-01-12

**Authors:** Eunji Mun, Kyung Hee Kim, Jung Eun Choi, Hyesook Park, Hye Ah Lee, Hae Soon Kim

**Affiliations:** 1 Department of Pediatrics, Soonchunhyang University Gumi Hospital, Gumi, Republic of Korea; 2 Department of Pediatrics, College of Medicine, Ewha Womans University, Seoul, Republic of Korea; 3 Department of Preventive Medicine, College of Medicine, Ewha Womans University, Seoul, Republic of Korea; 4 Graduate Program in System Health Science and Engineering, Ewha Womans University, Seoul, Republic of Korea; 5 Clinical Trial Center, Ewha Womans University Mokdong Hospital, Seoul, Republic of Korea

**Keywords:** Adolescent, Insulin resistance, Korea national health and nutrition examination survey, Vitamin d deficiency

## Abstract

**Objective:**

This study evaluated the relationship between vitamin D status and insulin resistance (IR) among Korean adolescents, focusing on the differences between normal-weight and overweight/obese groups using non-insulin-based IR indices.

**Methods:**

In this cross-sectional study, data from 3,838 adolescents (age: 12–18 years) who participated in the Korea National Health and Nutrition Examination Survey (KNHANES) from 2008 to 2014 were included. Using this nationally representative dataset, serum 25-hydroxyvitamin D (25(OH)D) levels were measured, and the prevalence of vitamin D deficiency (25(OH)D < 20 ng/mL) was estimated at the population level. The subjects were divided into the normal-weight and overweight/obese groups. We used the triglyceride–glucose index (TyG), triglyceride-to-high-density lipoprotein cholesterol ratio(TG/HDL-C), TyG with body mass index (TyG-BMI), and metabolic score for IR (METS-IR) as non-insulin-based IR indices. To evaluate the mean differences between groups and the associations with vitamin D status, we used a survey-weighted generalized linear regression model, adjusting for age, sex, household income, and strength training.

**Results:**

The prevalence of vitamin D deficiency among adolescents was 78.5%. In particular, the mean vitamin D levels were higher in boys, individuals who engaged in strength training, and individuals with waist circumferences below the 90th percentile. Vitamin D levels were significantly negatively associated with IR markers, particularly METS-IR in the normal-weight group and TyG-BMI and METS-IR in the overweight/obese group. The sensitivity analysis revealed that higher vitamin D levels were associated with a more substantial reduction in IR, especially in overweight/obese adolescents.

**Conclusions:**

Vitamin D deficiency is significantly associated with higher IR in adolescents, as measured by non-insulin-based indices. This association appears to be strong in overweight/obese individuals.

**Supplementary Information:**

The online version contains supplementary material available at 10.1186/s12887-026-06510-5.

## Introduction

Insulin resistance (IR) is a well-known risk factor for hyperglycemia, dyslipidemia, and hypertension, which are elements of metabolic syndrome (MetS) [1, 2]. In clinical settings, direct methods for measuring IR have limited practical application. Consequently, alternative indices, including the homeostasis model assessment of IR (HOMA-IR), are widely used [2, 3]. However, non-insulin-based IR markers can be calculated using readily available clinical values, making them more practical and cost-effective [4, 5]. Accordingly, recent studies have used non-insulin-based IR markers, including the triglyceride–glucose index (TyG), triglyceride-to-high-density lipoprotein cholesterol ratio (TG/HDL-C), TyG with body mass index (TyG-BMI), and metabolic score for IR (METS-IR) [6, 7]. In particular, the TyG is a reliable surrogate marker for IR, which performs better than HOMA-IR in adults with metabolic risk, patients with type 2 diabetes (BMI < 35 kg/m²), and adolescents [8–10].

Vitamin D plays an important role in various physiological processes (e.g., bone health, immune function, and metabolic regulation), and vitamin D deficiency increases the risk of cardiovascular disease, osteoporosis, and other chronic pathologies [11–14]. Higher vitamin D levels are associated with a lower risk of developing MetS [15]. However, vitamin D deficiency markedly increased in the Korean population between 2008 and 2014 [16]. According to previous studies, overweight and obese children are at a higher risk of vitamin D deficiency [17, 18].

Obesity is a well-known risk factor for IR and metabolic dysregulation in adolescents. Moreover, it may influence vitamin D metabolism. Excess adipose tissue can sequester vitamin D, reduce its bioavailability, and alter its endocrine functions, thereby exacerbating metabolic disturbances [19–21]. Subgrouping adolescents by weight status allows a more precise evaluation of the relationship between vitamin D and IR, helping to determine whether this association differs in normal-weight versus overweight/obese individuals. According to previous studies in children, vitamin D is inversely related to IR [22, 23], and this relationship may be more pronounced in individuals with higher BMI [24]. However, few studies have directly compared normal-weight and overweight/obese adolescents using non-insulin-based IR indices.

Therefore, the present study aimed to evaluate the relationship between vitamin D status and IR in Korean adolescents using non-insulin-based indices from the 2008–2014 Korea National Health and Nutrition Examination Survey (KNHANES), focusing on the differences between normal-weight and overweight/obese adolescents.

## Methods

### Data source and study subjects

This study was conducted using data from the 2008–2014 KNHANES, a nationally representative survey of the Korean population. KNHANES is a survey conducted annually by the Korea Centers for disease control and prevention to evaluate the health and nutritional status of Koreans. To collect data on various health-related indicators, trained staff conduct health interviews and health examinations and collect biochemical and clinical profiling data through blood and urine sampling. Datasets are publicly accessible via the official website of KNHANES (https://knhanes.kdca.go.kr). All participants consented to their data being used for research. Additional details about KNHANES can be obtained from other sources [25].

**Fig. 1:**
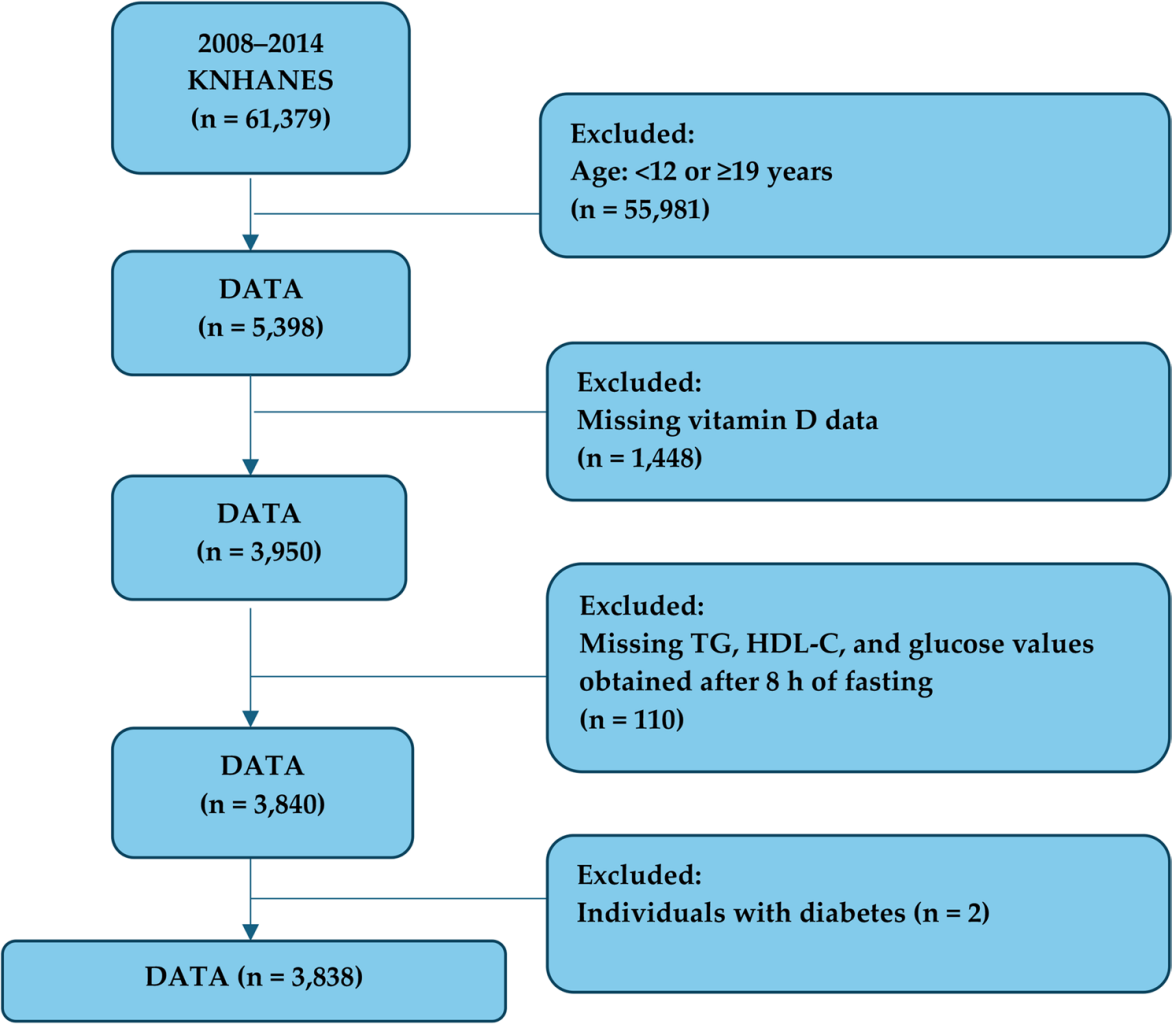
Flowchart of study subjects. Abbreviations: KNHANES, Korea National Health and Nutrition Examination Survey; TG, Triglyceride; HDL-C; High-density lipoprotein cholesterol

A total of 5,398 adolescents (age: 12–18 years) were selected from the KNHANES dataset. Participants with missing data on vitamin D, TG, HDL-C, and fasting blood glucose (FBG) levels; those who did not fast for 8 h; and those with diabetes were excluded from the study (*n* = 1,560). Consequently, 3,838 subjects (2,041 boys and 1,797 girls) were included in the final analysis (Fig. 1).

The publicly available KNHANES data did not contain any personal information. Therefore, the use of these data did not require ethical approval by the Institutional Review Board of Ewha Womans University Hospital (IRB no. 2025-02-027).

### Measurements

Height and weight were measured to the nearest 0.1 cm and 0.1 kg, respectively, with measurements taken barefoot and in light indoor clothing. The BMI was calculated as weight in kilograms divided by the square of height in meters. Participants with a BMI at the ≥ 85th percentile for age and sex were classified as overweight and obese [26] according to the 2017 Korean National Growth Charts for children and adolescents [27]. The waist circumference (WC) was measured at the smallest circumference between the lower edge of the rib cage and the iliac crest. The WC was analyzed by dividing the participants into groups based on whether it was above or below the 90th percentile [28].

The collected specimens were analyzed within 24 h at a certified laboratory. FBG, TG, and HDL-C levels were enzymatically measured using the Hitachi 7600 − 210 Automatic Analyzer (Hitachi, Tokyo, Japan). Serum 25-hydroxyvitamin D (25(OH)D) levels were determined using a 25-hydroxyvitamin D 125I radioimmunoassay kit (DiaSorin Inc., Stillwater, MN, USA) with a 1470 Wizard γ-counter (PerkinElmer, Turku, Finland). Vitamin D deficiency was defined as a serum 25(OH)D concentration of < 20 ng/mL [29, 30].

### Non-insulin-based IR indices

The TyG, TG/HDL-C, TyG-BMI, and METS-IR were included as non-insulin-based IR indices. The TyG was calculated using the following formula: log [fasting TG (mg/dL) × FBG (mg/dL)/2]. The TG/HDL-C was determined using the following formula: fasting TG (mg/dL)/HDL-C (mg/dL). Meanwhile, the TyG-BMI was obtained using the following formula: TyG × BMI. Finally, the METS-IR was calculated as follows: ln [(2 × FBG (mg/dL) + fasting TG (mg/dL)] × BMI)/ln [HDL-C (mg/dL)] [6, 7]. Higher values of these indices indicate greater IR.

### Covariates

Demographic and strength training variables were included as potential covariates. Household income was categorized into quartiles (low, medium-low, medium-high, and high) and calculated as the monthly household income divided by the square root of the household size. Meanwhile, muscle-strengthening exercise was defined as engaging in strength training at least two days per week [31, 32]. Physical activity was not included because of changes in the survey during the study period.

### Statistical analysis

All statistical analyses were performed using SAS version 9.4 (SAS Institute Inc., Cary, NC, USA), accounting for the survey’s sampling design. Specifically, PROC SURVEYMEANS and PROC SURVEYREG were used to account for the complex sampling design and sample weights of KNHANES. Aggregate weights were computed to generate appropriate statistics because multiyear datasets were used. Continuous variables that did not follow a normal distribution were log-transformed, and the results were presented after back-transformation [33]. Summary statistics were expressed as weighted means with 95% confidence intervals (CIs) for numeric data and number of subjects and weighted percentages for nominal data. The survey-weighted generalized linear regression model was used to assess the mean differences in the 25(OH)D concentrations across basic characteristics. We also estimated the correlation coefficients between non-insulin-based IR indices and vitamin D concentrations. Differences in non-insulin-based IR indices based on vitamin D deficiency (< 20 or ≥ 20 ng/mL) were assessed according to weight status (normal-weight vs. overweight/obese group). They were also evaluated by adjusting for sex, age, household income (continuous), and strength training (yes/no). As part of sensitivity analysis, vitamin D concentrations were assessed in more detailed groupings (< 20, 20–<30, and ≥ 30 ng/mL). In addition, the two-way interaction effect between obesity status and vitamin D levels on the non-insulin-based IR indices was examined. The threshold for statistical significance was set at *P* < 0.05 for two-tailed tests. For the interaction, *P* < 0.1 was considered statistically significant based on previous reports [34–37].

## Results

### Study participants’ baseline characteristics

Approximately 78.5% of participants were classified as having vitamin D deficiency (25(OH)D < 20 ng/mL), whereas only 21.5% had vitamin D levels of ≥ 20 ng/mL. Notably, only 1.7% of participants had vitamin D levels of ≥ 30 ng/mL (Table 1).

**Table 1:** Basic characteristics and 25-hydroxyvitamin D levels of study participants

	Total		25(OH)D (ng/mL)			
	*n*	Weighted %	Weighted mean	Lower 95%CI	Upper 95%CI	*P*-value
Overall	3838	100.00	15.39	15.12	15.66	
Age (years)						
12–15	2387	56.28	16.16	15.84	16.50	< 0.001
16–18	1451	43.72	14.44	14.10	14.79	
Sex						
Boys	2041	53.31	16.03	15.70	16.37	< 0.001
Girls	1797	46.69	14.68	14.36	15.01	
Household income						
Low	460	13.71	14.98	14.37	15.61	0.018
Medium-low	911	26.77	15.01	14.54	15.49	
Medium-high	1187	30.33	15.45	15.05	15.87	
High	1236	29.19	15.89	15.47	16.33	
Strength training						
Yes	941	24.79	16.54	16.09	17.01	< 0.001
No	2867	75.21	15.04	14.77	15.32	
Waist circumference (cm)						
WC (≥ 90th)	330	9.26	14.60	13.98	15.25	0.010
WC (< 90th)	3501	90.74	15.48	15.20	15.76	
BMI (kg/m^2^)						
Overweight and obesity (≥ 85th)	739	19.96	15.07	14.62	15.53	0.099
Normal (< 85th)	3094	80.04	15.47	15.18	15.77	
25(OH)D (ng/mL)						
<10ng/mL	357	10.05	8.57	8.41	8.73	< 0.001
10–<20ng/mL	2628	68.42	14.63	14.47	14.78	
20–<30ng/mL	789	19.83	23.11	22.89	23.33	
≥30ng/mL	64	1.71	32.93	32.29	33.58	

The values represent weighted means and 95% CIs for continuous variables and frequencies and weighted percentages for categorical variables

*Abbreviations*: *25(OH)D* 25-hydroxyvitamin D, *CI* Confidence interval, *BMI* Body mass index, *WC* Waist circumference

The mean vitamin D level of adolescents aged 12–15 years was 16.16 ng/mL, which was higher than the 14.44 ng/mL observed in those aged 16–18 years. In addition, boys had a mean vitamin D level of 16.03 ng/mL, which was higher than that observed in girls (14.68 ng/mL). Vitamin D levels were also higher in individuals who engaged in strength training (16.54 ng/mL) than in those who did not (15.04 ng/mL). Moreover, individuals with a WC below the 90th percentile had a mean vitamin D level of 15.48 ng/mL, whereas those with a WC above the 90th percentile had a mean vitamin D level of 14.60 ng/mL. Adolescents classified as overweight or obese (BMI ≥ 85th percentile) had lower vitamin D levels than normal-weight adolescents. However, the difference was not statistically significant.

In the comparison between vitamin-D-deficient and nondeficient groups, vitamin D deficiency was more common in older adolescents (16–18 years) and those with a WC ≥ 90th percentile or BMI ≥ 85th percentile. By contrast, boys and individuals engaging in strength training were more likely to be nondeficient (Supplemental Table 1).

### Correlation coefficients of non-insulin-based IR indices with vitamin D concentration

In examining the correlation coefficients between non-insulin-based IR indices and vitamin D, a negative correlation was observed between BMI and 25(OH)D. In addition, the TyG-BMI and METS-IR showed a negative correlation with 25(OH)D. Each non-insulin-based IR index had a strong positive correlation with BMI, and a strong positive correlation was observed between the non-insulin-based IR indices themselves (Table 2).

**Table 2:** Correlation coefficients of non-insulin-based insulin resistance indices with vitamin D concentration

	Weighted mean	95% CI	Correlation coefficient (γ)				
			25(OH)D	BMI	TG/HDL-C	TyG	TyG-BMI
BMI, kg/m^2^	21.23	21.07–21.38	−0.070**				
TG/HDL-C	1.51	1.47 − 1.55	0.000	0.293***			
TyG	4.40	4.39 − 4.41	−0.010	0.245***	0.940***		
TyG-BMI	91.93	91.24 − 92.63	−0.065**	0.951***	0.540***	0.515***	
METS-IR	29.88	29.63 − 30.12	−0.055*	0.944***	0.533***	0.433***	0.968***

*Abbreviations*: *95% CI* 95% confidence interval, *25(OH)D* 25-hydroxyvitamin D, *BMI* Body mass index, *TG/HDL-C* Triglyceride-to-high-density lipoprotein cholesterol ratio, *TyG* Triglyceride–glucose index, *TyG-BMI* TyG with body mass index, *METS-IR* metabolic score for insulin resistance

*P < 0.05, ** *P* < 0.01, and *** *P* < 0.001

### Association between vitamin D and IR by weight status

The relationship between vitamin D level and IR was separately analyzed in the normal-weight and overweight/obese groups (Table 3).

**Table 3:** Association between 25(OH)D level and non-insulin-based insulin resistance indices by weight status

		Unadjusted model								Adjusted model ^a^							
		Normal weight				Overweight and Obesity				Normal weight				Overweight and Obesity			
Outcome	25(OH)D (ng/mL)	Weighted mean	Lower 95% CI	Upper 95%CI	*P*-value	Weighted mean	Lower 95%CI	Upper 95%CI	*P*-value	Weighted mean	Lower 95%CI	Upper 95%CI	*P*- value	Weighted mean	Lower 95%CI	Upper 95%CI	*P*-value
TG/HDL-C	< 20	1.39	1.36	1.43	0.924	2.09	1.96	2.24	0.437	1.39	1.35	1.43	0.975	2.13	1.99	2.28	0.247
	≥ 20	1.39	1.32	1.47		1.96	1.69	2.28		1.39	1.32	1.47		1.93	1.67	2.24	
TyG	< 20	4.37	4.36	4.38	0.845	4.52	4.49	4.55	0.295	4.37	4.36	4.38	0.736	4.53	4.50	4.56	0.169
	≥ 20	4.37	4.35	4.40		4.48	4.42	4.55		4.37	4.35	4.40		4.48	4.41	4.54	
TyG-BMI	< 20	86.38	85.83	86.93	0.014	120.72	119.00	122.49	0.023	86.02	85.48	86.56	0.095	120.57	118.93	122.22	0.019
	≥ 20	84.80	83.69	85.94		116.70	113.84	119.64		84.93	83.79	86.08		116.55	113.73	119.44	
METS-IR	< 20	27.97	27.78	28.16	0.014	39.80	39.20	40.42	0.056	27.87	27.68	28.06	0.036	39.77	39.19	40.35	0.034
	≥ 20	27.45	27.09	27.82		38.61	37.60	39.64		27.42	27.05	27.79		38.51	37.55	39.50	

*Abbreviations*: *25(OH)D* 25-hydroxyvitamin D, *TG/HDL-C* Triglyceride-to-high-density lipoprotein cholesterol ratio, *TyG* Triglyceride–glucose index, *TyG-BMI* TyG with body mass index, *METS-IR* Metabolic score for insulin resistance

^a^Adjusted for sex, age, household income, and strength training

In the normal-weight group, the TyG-BMI was inversely related to vitamin D levels, with higher values being observed in the group with lower vitamin D levels (*P* = 0.014). However, this association was no longer statistically significant after adjustment (*P* = 0.095). Meanwhile, the negative association between the METS-IR and vitamin D levels was maintained even after adjustment (*P* = 0.036).

In the overweight/obese group, the TyG-BMI was also inversely related to vitamin D levels (*P* = 0.023), and this relationship remained statistically significant even after adjustment (*P* = 0.019). For the METS-IR, the inverse relationship with vitamin D levels was confirmed after adjustment (*P* = 0.034).

The results of analyses performed using the TG/HDL-C and TyG showed that lower vitamin D levels in the overweight/obese group were associated with higher IR. However, these relationships were not statistically significant.

Overall, the mean IR difference between the vitamin D groups was generally larger in the overweight/obese group than in the normal-weight group for all IR indices. In the METS-IR, the normal-weight group had IR values of 27.87 and 27.42 in the vitamin D < 20 ng/mL and vitamin D ≥ 20 ng/mL groups, respectively, with a difference of 0.45 in the adjusted model. In the overweight/obese group, the METS-IR difference was 1.26, indicating a larger disparity in IR values compared with that in the normal-weight group. This trend was consistently observed across all IR indicators.

### Interaction of vitamin D levels and overweight/obesity status with the TG/HDL-C and TyG

**Fig. 2:**
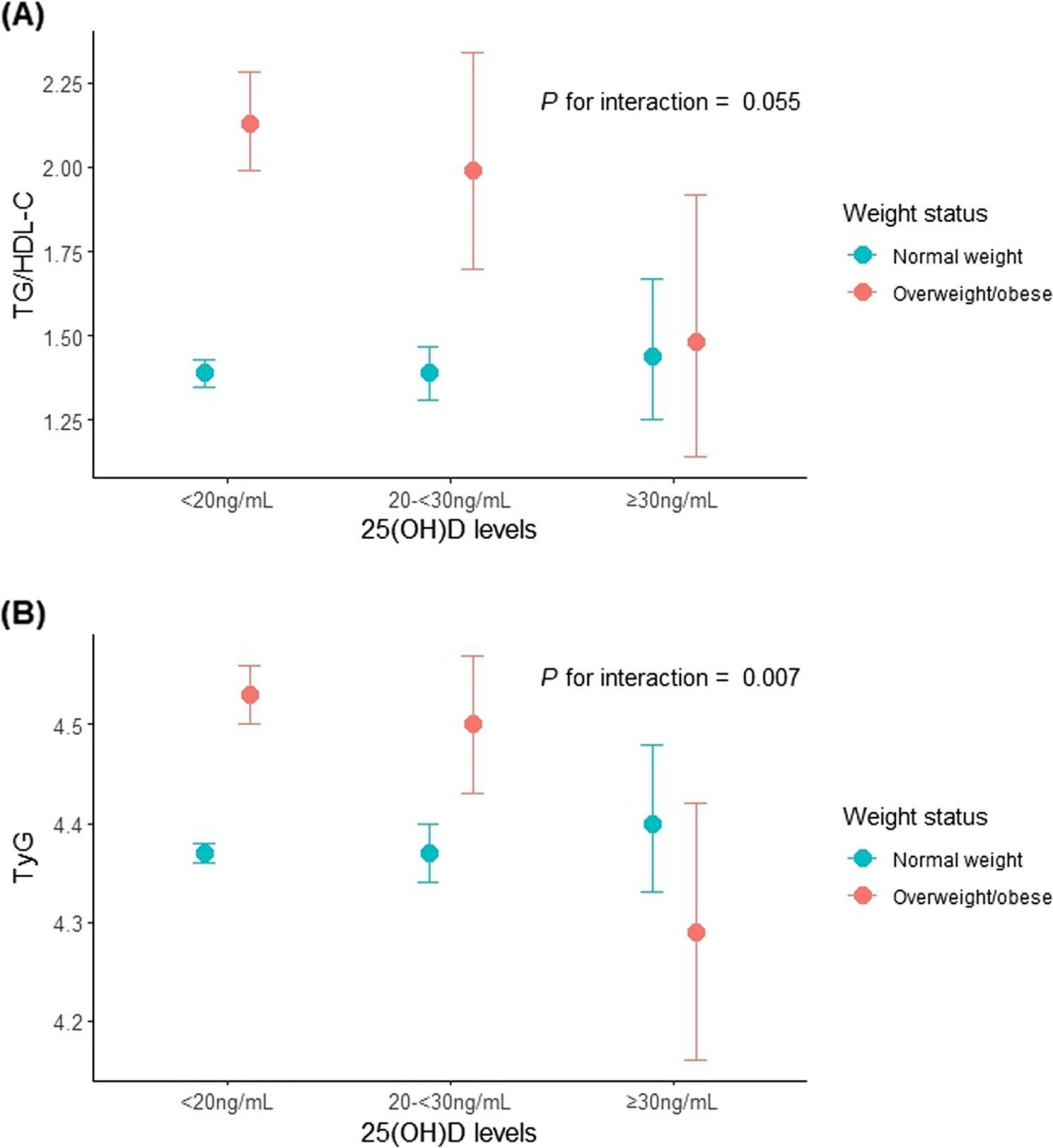
Interaction between vitamin D levels and overweight/obesesity status on insulin resistance. (A) Interaction between vitamin D levels and weight status on TG/HDL-C. (B) Interaction between vitamin D levels and weight status on TyG.Values represent the adjusted means and 95% confidence intervals after adjusting for sex, age, household income, and strength training and were estimated via the survey-weighted generalized linear regression model. Abbreviations: TG/HDL-C, triglyceride-to-high-density lipoprㅁotein cholesterol ratio; TyG, triglyceride–glucose index; 25(OH)D, 25-hydroxyvitamin D

When vitamin D levels were stratified into three groups (< 20, 20–<30, and ≥ 30 ng/mL), an interaction effect between vitamin D levels and overweight/obesity status was observed for the TG/HDL-C (Pinteraction = 0.055) and TyG (Pinteraction = 0.007) (Fig. 2).

Higher levels of vitamin D were linked to a more significant decrease in IR, especially in the overweight/obese group. Notably, IR in the overweight/obese group approached or even fell below that of the normal-weight group when vitamin D levels were high.

## Discussion

### Prevalence of vitamin D deficiency among Korean adolescents

In the present study, approximately 78.5% of Korean adolescents were classified as vitamin D deficient, whereas only 1.7% had vitamin D levels ≥ 30 ng/mL. These rates are considerably higher than those reported in other countries, including 42.0% of adolescents in the United States and 12.5% of children and adolescents in Brazil [38, 39]. These findings underscore the need for public health interventions, including dietary supplementation, promotion of outdoor activities, and vitamin D fortification programs, to improve the vitamin D status among adolescents.

### Effects of obesity on the relationship between vitamin D status and IR

Consistent with previous studies, our findings demonstrated an inverse association between vitamin D levels and IR indices (TyG-BMI and METS-IR) [22, 23]. In the normal-weight group, the association between vitamin D and TyG-BMI weakened after adjusting for confounders. By contrast, in the overweight/obese group, the TyG-BMI and METS-IR maintained their negative association with vitamin D levels after adjustment, indicating a more robust relationship. In a meta-analysis in Nutrients (2021), serum vitamin D levels were inversely associated with HOMA-IR. Moreover, the strength of this relationship progressively increased from the lowest (< 25 kg/m²) to the highest (> 30 kg/m²) BMI quartile, indicating a stronger link between vitamin D deficiency and IR in individuals with higher BMI [40]. Vitamin D is sequestered in adipose tissue in overweight and obese adolescents, leading to lower circulating levels. This deficiency may exacerbate IR by impairing insulin sensitivity and promoting inflammation. Moreover, vitamin D deficiency could disrupt pancreatic β-cell function and insulin secretion, particularly in the context of obesity-related chronic inflammation [19–21]. These findings underscore the potential importance of maintaining adequate vitamin D status, particularly in overweight or obese adolescents, as a strategy to mitigate metabolic risk.

### Implications of vitamin D supplementation

In our sensitivity analysis, overweight/obese adolescents with higher vitamin D levels (≥ 30 ng/mL) showed similar, or even lower, IR indices to the normal-weight group. This finding raises the possibility that optimizing vitamin D status could play a beneficial role in improving metabolic health, particularly among adolescents at a higher risk of IR because of excess weight. Although causality cannot be established from our study, these results suggest that vitamin D supplementation or increased sun exposure might be a potential intervention strategy to mitigate IR in overweight/obese adolescents.

According to evidence from interventional studies, vitamin D supplementation has potential metabolic benefits. For example, a double-blind clinical trial involving overweight and obese women aged 20–40 years found that vitamin D supplementation (50,000 IU/week) for six weeks significantly reduced BMI, body weight, and WC [41]. This finding suggests that high-dose vitamin D supplementation can decrease leptin levels and ameliorate glucose metabolism, particularly in insulin-resistant, overweight, or obese individuals [41, 42].

However, randomized clinical trials have reported inconsistent results regarding the efficacy of vitamin D in managing altered metabolic states [42]. Further studies are needed to clarify the role of vitamin D supplementation in glucose metabolism and its therapeutic potential in addressing IR and related metabolic disorders.

### Limitations

This study has some limitations that should be acknowledged. First, this study identified associations but could not establish causality between vitamin D and IR because of its cross-sectional design. Second, the study’s limited scope (focusing on a single ethnicity) might have reduced the findings’ generalizability to more ethnic groups. Third, the questionnaire items were limited because of the survey’s multiyear nature. Consequently, only strength training could be included under physical activity. Factors such as seasonal variation or supplement use could not be considered. Thus, residual confounding factors might have influenced the study findings.

In addition, we focused on the categorical thresholds of vitamin D deficiency (< 20 ng/mL) rather than modeling the continuous dose–response relationships. This choice was made because categorical cut-offs are clinically meaningful and widely used in pediatric and adolescent populations and they facilitated subgroup comparisons by obesity status. Nonetheless, we recognize that spline-based or other continuous modeling approaches could help clarify potential nonlinear associations. This should be addressed in future research. Finally, subgroup analyses were separately performed for normal-weight and overweight/obese participants, which involved stratification rather than multiple simultaneous pairwise testing. Therefore, no formal correction for multiple comparisons was applied. However, the possibility of a type I error cannot be excluded, and the results should be interpreted with appropriate caution.

### Clinical and public health implications

This study investigated the relationship between vitamin D levels and IR in adolescents using a robust and nationally representative dataset, focusing on weight status. Several recently proposed non-insulin-based IR indices were used to assess IR. By analyzing the association between vitamin D and IR according to weight status, these findings suggest that addressing vitamin D deficiency could be a promising strategy for improving metabolic health, particularly in overweight and obese adolescents. However, future studies are needed to confirm whether correcting vitamin D deficiency in this population leads to significant improvements in IR and long-term metabolic outcomes.

## Conclusion

This study highlights a significant association between vitamin D levels and IR using non-insulin-based IR markers in adolescents, with notable differences being observed between the normal-weight and overweight/obese groups. These findings underscore the importance of vitamin D in IR management, especially in overweight or obese adolescents.

## Supplementary Information

Supplementary Material 1.

## Data Availability

No datasets were generated or analysed during the current study.

## Abbreviations

IR: Insulin resistance
25(OH)D: 25-hydroxyvitamin D
TyG: Triglyceride-glucose index
TG/HDL-C: Triglyceride-to-high-density lipoprotein cholesterol ratio
TyG-BMI: Triglyceride-glucose index with body mass index
METS-IR: Metabolic score for insulin resistance
MetS: Metabolic syndrome
HOMA-IR: Homeostasis model assessment of insulin resistance
T2DM: Type 2 diabetes mellitus
KNHANES: Korea National Health and Nutrition Examination Survey
FBG: Fasting blood glucose
BMI: Body mass index
WC: Waist circumference
TG: Triglycerides
HDL-C: High-density lipoprotein cholesterol
CI: Confidence interval
