# Supplementary material for: Association between vitamin D status and insulin resistance in Korean adolescents: differential effects of obesity using non-insulin-based indices
Source: BMC Pediatr. 2026 Jan 12;26:140. doi: 10.1186/s12887-026-06510-5 (PMC12918070; doi:10.1186/s12887-026-06510-5)
Supplement: Supplementary file 1 — Supplementary Material 1. [file 12887_2026_6510_MOESM1_ESM.docx]

**Supplementary Materials**

Supplemental Table 1. Comparison of covariates between vitamin-D-deficient and nondeficient groups

|  | **25(OH)D (ng/mL)** | | | |  | |
| --- | --- | --- | --- | --- | --- | --- |
|  | **< 20** | | **≥ 20** | | | ***P*-value** |
|  | **n** | **Weighted %** | **n** | **Weighted %** | |  |
| **Overall** | 2985 | 78.47 | 853 | 21.53 | |  |
| **Age (years)** |  |  |  |  | |  |
| **12–15** | 1765 | 52.87 | 622 | 68.70 | | < 0.001 |
| **16–18** | 1220 | 47.13 | 231 | 31.30 | |  |
| **Sex** |  |  |  |  | |  |
| **Boys** | 1514 | 51.10 | 527 | 61.37 | | < 0.001 |
| **Girls** | 1471 | 48.90 | 326 | 38.63 | |  |
| **Household income** |  |  |  |  | |  |
| **Low** | 365 | 13.97 | 95 | 12.75 | | 0.177 |
| **Medium-low** | 721 | 27.29 | 190 | 24.84 | |  |
| **Medium-high** | 921 | 30.62 | 266 | 29.27 | |  |
| **High** | 945 | 28.11 | 291 | 33.14 | |  |
| **Strength training** |  |  |  |  | |  |
| **No** | 2301 | 77.53 | 566 | 66.74 | | < 0.001 |
| **Yes** | 663 | 22.47 | 278 | 33.26 | |  |
| **Waist circumference (cm)** |  |  |  |  | |  |
| **WC (<90th)** | 2697 | 89.88 | 804 | 93.88 | | 0.010 |
| **WC (≥90th)** | 281 | 10.12 | 49 | 6.12 | |  |
| **BMI (kg/m^2^)** |  |  |  |  | |  |
| **Normal (<85th)** | 2380 | 79.00 | 714 | 83.84 | | 0.010 |
| **Overweight and obesity (≥85th)** | 602 | 21.00 | 137 | 16.16 | |  |

Abbreviations: 25(OH)D, 25-hydroxyvitamin D; BMI, body mass index; WC, waist circumference.

The values represent frequencies and weighted percentages. *P*-values were obtained from the Rao–Scott chi-square test
